# RESULTS FROM A MIXED METHODS RANDOMIZED CONTROLLED TRIAL: PEER SUPPORT FOR DEPRESSION AMONG VULNERABLE OLDER ADULTS

**DOI:** 10.1093/geroni/igae098.0875

**Published:** 2024-12-31

**Authors:** Jinhui Joo

**Affiliations:** Massachusetts General Hospital, Boston, Massachusetts, United States

## Abstract

Depressed, low-income White and BIPOC older adults do not access mental health services despite increased risk. We evaluated the impact of a one-to-one peer support intervention delivered by older adult peer mentors compared to an active social control delivered by non-peers. The primary outcome was depression with up to 12 months follow up. Mediation analysis and post-study interviews were conducted with both groups. Among 149 randomized participants, the mean age was 70, 84% were women, 52% were Black and 41% were White. Both groups experienced an average decrease of 2.54 (SD: 5.80) points in PHQ-9 scores compared to their baseline levels that was sustained at 12 months. Emotional well-being, and self-efficacy increased, and adaptive coping, and loneliness decreased during the intervention and at follow up for both groups. Loneliness was a mediator for both groups but the intervention selectively engaged self-efficacy and adaptive coping. Qualitative data suggest that the intervention and control groups had disparate experiences. The intervention group learned coping skills and enacted behavior change, whereas the control group did not. Our results indicate that the PEERS intervention was not superior to social interactions delivered by non-peers, but the mechanism may be different. The intervention may increase active coping and self-efficacy whereas social interaction provided by non-peers decreased loneliness. Lay older adult peer mentors can be trained and impact important factors related to well being. Increased structure in peer support content may be needed to observe impact on depression outcomes.

